# Carbon-based single-atom catalysts in advanced oxidation reactions for water remediation: From materials to reaction pathways

**DOI:** 10.1016/j.eehl.2023.04.002

**Published:** 2023-04-25

**Authors:** Junjie Zhang, Xu Tang, Yongjia Hong, Guanyu Chen, Yong Chen, Li Zhang, Wenran Gao, Yang Zhou, Bin Sun

**Affiliations:** a State Key Laboratory of Organic Electronics and Information Displays, Institute of Advanced Materials (IAM), School of Material Science and Engineering, Nanjing University of Posts and Telecommunications, Nanjing 210023, China; b Joint International Research Laboratory of Biomass Energy and Materials, Co-Innovation Center of Efficient Processing and Utilization of Forest Resources, College of Materials Science and Engineering, Nanjing Forestry University, Nanjing 210037, China

**Keywords:** Single-atom catalysts, Emerging contaminants, Advanced oxidation processes, Persulfate activation, Reactive oxidant species

## Abstract

Single-atom catalysts (SACs) have been widely recognized as state-of-the-art catalysts in environment remediation because of their exceptional performance, 100% metal atomic utilization, almost no secondary pollution, and robust structures. Most recently, the activation of persulfate with carbon-based SACs in advanced oxidation processes (AOPs) raises tremendous interest in the degradation of emerging contaminants in wastewater, owning to its efficient and versatile reactive oxidant species (ROS) generation. However, the comprehensive and critical review unraveling the underlying relationship between structures of carbon-based SACs and the corresponding generated ROS is still rare. Herein, we systematically summarize the fundamental understandings and intrinsic mechanisms between single metal atom active sites and produced ROS during AOPs. The types of emerging contaminants are firstly elaborated, presenting the prior pollutants that need to be degraded. Then, the preparation and characterization methods of carbon-based SACs are overviewed. The underlying material structure–ROS type relationship in persulfate-based AOPs is discussed in depth to expound the catalytic mechanisms. Finally, we briefly conclude the current development of carbon-based SACs in AOPs and propose the prospects for rational design and synthesis of carbon-based SACs with on-demand catalytic performances in AOPs in future research.

## Highlights

• Persulfate activation mechanism is expounded in terms of material structure–reactive oxidant species type relationship.
• Design and synthesis protocols of carbon-based single-atom catalysts with on-demand catalytic performances are proposed.
• The preparation and characterization strategies of carbon-based single-atom catalysts are overviewed.
• The prior emerging contaminants that need to be degraded are elaborated.

## Graphical abstract

**Figure:**
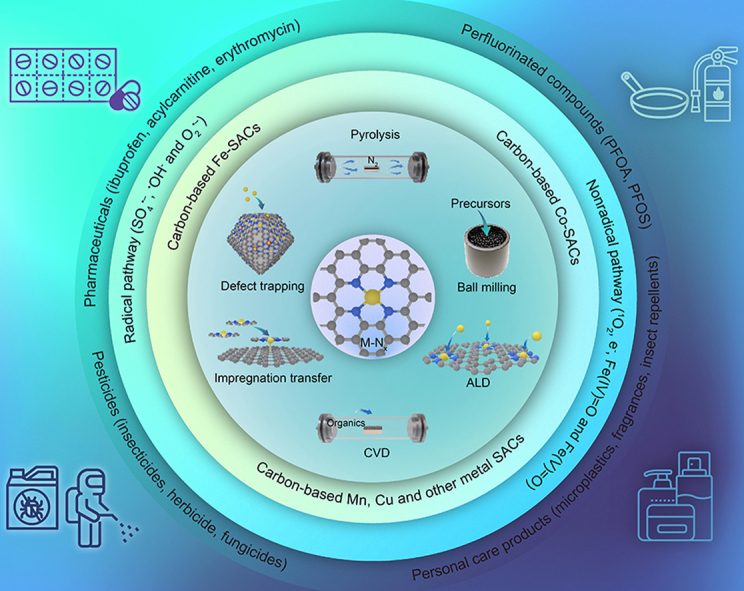

## 1 Introduction

Water quality is commonly evaluated by investigating its contents of inorganic pollutants, organic pollutants, microbial pollutants, and heavy metals [[1], [2], [3], [4]]. However, recent research reported some unknown pollutants, termed emerging contaminants, that are not legislatively listed as harmful to the environment [5]. We have gained little recognition of the emerging contaminants, as they appear seldom [6,7]. Although the emerging contaminants typically exist in concentrations that range from ng/L to μg/L, they are refractory to be degraded under natural conditions and accumulate in macroinvertebrates through the food chain [8]. Their release into the environment, therefore, has put a huge threat to human health and the environment [9,10]. To remove these emerging contaminants, various treatment methods have been developed, including phase-changing technologies [11], biological processes [12], and advanced oxidation processes (AOPs) [13]. Among them, AOPs show the greatest potential because of their highly efficient generation of reactive oxidant species (ROS), which mainly consist of hydroxyl radical (^•^OH), sulfate radicals (SO_4_^•-^), superoxide radical (^•^O_2_^-^), singlet oxygen (^1^O_2_), and high-valent metals-oxo species [[14], [15], [16]]. These ROS possess high oxidation potential, which can thus effectively attack and degrade emerging contaminants [3,17]. Due to the higher oxidizing capability and wider pH operational window of SO_4_^•-^ (E^θ^ = 2.5–3.1 V) than ^•^OH (E^θ^ = 1.8–2.7 V), persulfate-based AOPs, including peroxydisulfate (PDS) and peroxymonosulfate (PMS) activation have recently been the hotspot in water treatment [18,19].

**Table 1:** Mechanism of ROS generation at different single-atom active sites.

ROS	Active site	Mechanism	Pollutant	Oxidant	Ref.
SO_4_^•-^, ^•^OH	Fe-N_x_	Breakage of O–O	Phenol	PMS	[113]
SO_4_^•-^, ^•^OH	Fe(II)-pyridinic N_4_	Breakage of O–O	Bisphenol A	PMS	[114,115]
SO_4_^•-^, ^•^OH	Fe(III)-pyrrolic N_4_	Breakage of O–O	Bisphenol A	PMS	[36]
SO_4_^•-^, ^•^OH	Fe–N_4_	Breakage of O–O	Bisphenol A	PMS	[116]
SO_4_^•-^,^•^OH	Fe–N_4_	Breakage of O–O	P-nitrophenol	PMS	[117]
SO_4_^•-^, ^•^OH	Fe–N_5_	Breakage of O–O	Sulfamethoxazole	PMS	[121]
SO_4_^•-^, ^•^OH	Co–N_4_	Breakage of O–O	Bisphenol A	PMS	[124,125]
SO_4_^•-^, ^•^OH	Co–N_3_	Breakage of O–O	Bisphenol A	PDS	[126]
SO_4_^•-^, ^•^OH	Co–O_2_	Breakage of O–O	1,4-dioxane	PMS	[127]
SO_4_^•-^, ^•^OH	Mn–N_4_	Breakage of O–O	Bisphenol A	PMS	[129]
^1^O_2_	Fe–N_4_	SO_5_^•-^ self-reaction	Bisphenol A	PMS	[135]
^1^O_2_	Fe–N_4_	SO_5_^•-^ self-reaction	P-chlorophenol	PMS	[134]
^1^O_2_	Fe-pyridine N_4_	SO_5_^•-^ self-reaction	Sulfasalazine	PMS	[137]
^1^O_2_	Co–N_4_B_2_	SO_5_^•-^ self-reaction	Sulfamethazine	PMS	[151]
^1^O_2_	Co–N_4_	SO_5_^•-^ self-reaction	Bisphenol A	PMS	[35,131,152]
^1^O_2_	Co–N_3_P	SO_5_^•-^ self-reaction	Sulfadiazine	PMS	[153]
^1^O_2_	Co–N_4_	SO_5_^•-^ self-reaction	Phenol	PMS	[154]
^1^O_2_	Co–N_4_	SO_5_^•-^ self-reaction	17β-estradiol	PMS	[155]
^1^O_2_	Co–N_2+2_	SO_5_^•-^ self-reaction	Ciprofloxacin	PMS	[106]
^1^O_2_	Co–N_3_Si_1_	SO_5_^•-^ self-reaction	Bisphenol A	PMS	[159]
^1^O_2_	Mn–N_4_	SO_5_^•-^ self-reaction	Enrofloxacin	PMS	[109]
^1^O_2_	Ru–N_4_	SO_5_^•-^ self-reaction	Orange II	PMS	[105]
^1^O_2_	Cu–N_4_	SO_5_^•-^ self-reaction	Bisphenol A	PMS	[162]
^1^O_2_	Cu–N_4_	SO_5_^•-^ self-reaction	Bisphenol A	PDS	[119]
^1^O_2_	Fe–N_4_	^•^O_2_^-^ recombination	Phenol	PMS	[130]
^1^O_2_	Fe–N_4_	^•^O_2_^-^ recombination	Tetracycline	PMS	[136]
^1^O_2_	Fe–N_4_	OH^a^ evolution	Bisphenol A	PMS	[133]
^1^O_2_	Fe–N_4_	O_2_ activation	Tetracycline	PMS	[146]
^1^O_2_	Co–N_3_O_1_	OH^a^ evolution	Ciprofloxacin	PMS	[156]
^1^O_2_	Co–N_4_	HOO^a^ evolution	Bisphenol A	PMS	[157]
e^-^	Fe–N_4_	Electron transfer	O-phenylphenol	PMS	[140]
e^-^	Fe–N_4_O_1_	Electron transfer	Bisphenol A	PMS	[147]
e^-^	Fe-N_x_	Electron transfer	Bisphenol A	PMS	[141]
e^-^	Co–N_4_	Electron transfer	Sulfamethoxazole	PMS	[132]
e^-^	Co–N_4_	Electron transfer	Chloroquine phosphate	PMS	[160]
Fe(IV)=O	Fe(II)–N_4_	Fe(IV)=O	Phenol	PMS	[142]
Fe(IV)=O	Fe(II)–N_4_	Fe(IV)=O	Bisphenol A	PMS	[143]
Fe(IV)=O	Fe(II)–N_4_	Fe(IV)=O	Bisphenol A	PMS	[148]
Fe(V)=O	Fe(III)–N_4_	Fe(V)=O	P-chlorophenol	PMS	[144]
Fe(IV)=O	Fe(III)–N_4_	Fe(V)=O	2,4-dichlorophenol	PDS	[145]
Co(IV)	Co–N_3_	Co(IV)	Rhodamine B	PMS	[161]

a All the catalysts are prepared by pyrolysis strategy, expect for ref. [127] in which the catalyst is prepared by impregnation transfer.

Previously, heterogeneous metal nanoparticles or atomic clusters were the most effective catalysts applied in persulfate-based AOPs, due to the high electron-donating ability of metals [20,21]. However, these catalysts encounter the problem of poor stability, poor recyclability, secondary pollution, and low metal atoms utilization efficiency [22]. In contrast, single-atom catalysts (SACs) have been regarded as superior alternatives for traditional metal catalysts, given their robust structure, superior stability, 100% atomic utilization, and special electronic structure [[23], [24], [25]]. The concept of SACs was firstly proposed by Zhang et al., as they downsized the metal catalysts to an atomic level and prepared single Pt atoms anchored FeO_x_ nanocrystallites [26]. The single Pt atoms catalysts exhibited three-time enhancement in CO oxidation than bulk Pt, owing to their special electronic structure. Numerous strategies are developed to prepare SACs [27]. The principle for SACs preparation is anchoring and stabilizing single metal atoms on a suitable carrier, preventing its migration and aggregation [28]. Based on these principles, pyrolysis, defect/vacancy trapping, and other strategies have been developed to anchor single metal atoms on carriers such as metals, metal compounds, layered double hydroxides, and carbon materials. As a result, Fe, Cu, Co, Pt, Au, Rh, Pd, and other transition/noble metal atoms SACs anchored on various metal/metal-free carriers are reported. These SACs are widely applied in energy storage, energy conversion, electrocatalysis, biology, and environment [[29], [30], [31], [32], [33], [34]].

Until 2018, single cobalt atoms anchored on porous N-doped graphene were initially utilized as a highly reactive catalyst for PMS activation to degrade bisphenol A [35]. The single Co atom was demonstrated to show the optimal binding energy with PMS, enhancing the ^1^O_2_ generation produced from PMS activation. To date, significant progress has been made in the application of SACs in persulfate-based AOPs [[36], [37], [38], [39]]. Despite the encouraging development of SACs in persulfate-based AOPs, the application of SACs in the degradation of emerging contaminants by persulfate-based AOPs is still in its infancy [40]. As far as we are concerned, recent reviews mainly concentrated on the concept, preparation, and characterization of SACs [[41], [42], [43], [44], [45]]. The discussion and comprehension regarding the catalytic mechanisms are still far lagging behind. There is no relevant review that emphasizes the relationship between catalytic mechanism and structure of single-atom active sites in terms of the essential ROS generation. Considering the uniformity of active sites distributed in carbon-based SACs and metal-free compositions of carbon carriers [46,47], as well as the fact that recent research is primarily limited to carbon-based SACs, we select carbon-based SACs as the representative SACs in our discussion.

**Fig. 1:**
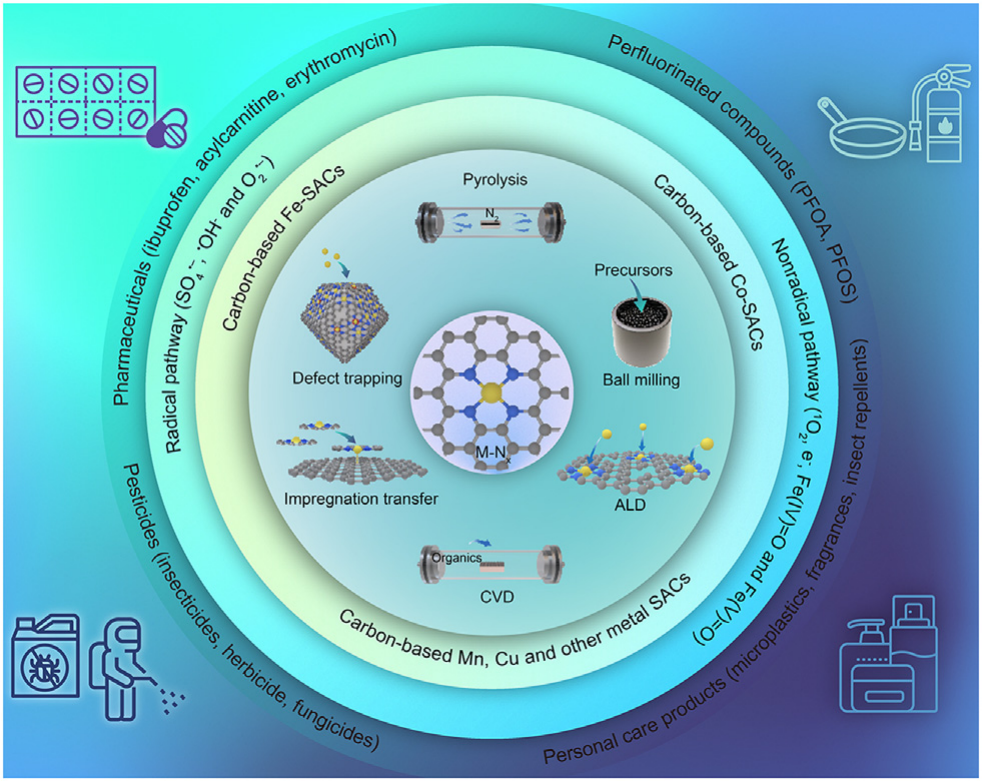
Overview of the topics discussed in this review.

In this review, we aim to summarize recent progress on the breakthrough of carbon-based SACs in persulfate-based AOPs to degrade emerging contaminants, as illustrated in Fig. 1. First, the types of emerging contaminants are clarified and corresponding typical contaminants are presented. Second, this article summarizes the progresses in the synthesis and characterization of carbon-based SACs in recent years. Third, the relationship between types of generated ROS in persulfate activation and the underlying single-atom active sites of carbon-based SACs are systematically summarized and discussed. Finally, we highlight brief conclusions and perspectives for the design of carbon-based SACs with controllable active sites toward targeted catalytic properties in persulfate-based AOPs from design principle, synthesis strategy, and distinction of single metal active sites.

## 2 Types of emerging contaminants

**Fig. 2:**
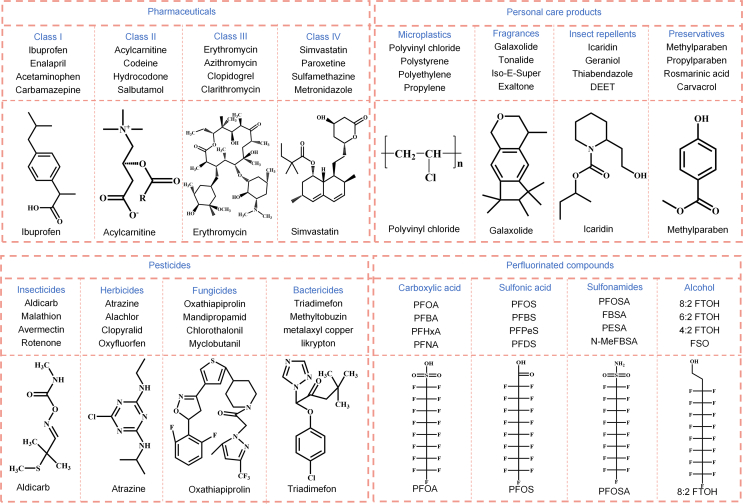
Classification and representative structures of emerging contaminants.

Emerging contaminants can be defined as any newly detected and identified natural, synthetic, or biological chemicals that are poorly or even unregulated in the environment but have adverse impacts on human health and ecosystems [8,48]. These contaminants are ubiquitous in the ecosystems and pose a potential risk to human beings and biodiversity even at trace amount levels [49]. Removing these emerging contaminants is, thus, urgently imperative. As showcased in Fig. 2, the emerging contaminants can mainly be divided into four categories: pharmaceuticals, personal care products, pesticides, and perfluorinated compounds from their industrial use point.

### 2.1 Pharmaceuticals

Pharmaceuticals are designed chemical compounds that minimize the sicknesses of human beings and livestock, as well as to increase the attachment value for human and animal life [50]. They have been widely utilized in different classes of chemicals such as nutrition, diagnosis, remedy, and preventative medicine [51]. Due to their structure versatility, more than 13,336 drugs are recorded in the DrugBank database 2019 (version 5.1.3, released on April 2, 2019) [52]. Nowadays, the global annual drug consumption ranges from 100,000 to 200,000 tons [53], and most of these drugs can be administered orally or by injection. However, pharmaceuticals possess stable chemical structures, making them less possible to be completely assimilated in the body or metabolized in the environment. Consequently, they can escape into the environment and distribute in waste water, ground water, surface water, and even in tap water [54].

The pharmaceuticals detected in aquatic environments can specifically be categorized into four classes based on their fate in water [5,55]. For the first class, it is almost totally removed after treatment (removal efficiency >95%), such as ibuprofen, enalapril, acetaminophen, and carbamazepine. For the second class, it cannot be completely removed after treatment, including acylcarnitine, codeine, hydrocodone, and salbutamol [56]. For the third class, it cannot be found in influential effluent but instead is detected in sewage water of effluents, for example, erythromycin, azithromycin, clopidogrel, and clarithromycin [57]. For the fourth class, it has never been identified in influential or wastewater effluent, such as simvastatin, paroxetine, sulfamethazine, and metronidazole. Exposure to pharmaceuticals and corresponding metabolites through food or water is believed to have short- and long-term impacts on human and aquatic species [58]. It could cause endocrine system disruptions, chronic toxicity, and drug-resistant bacterial strains increment.

### 2.2 Personal care products

Personal care products, including perfumes, preservatives, and sun screens, are utilized by people and animals for beauty, cleaning, and personal health [59]. They consist of a large number of prescribed and nonprescribed pharmaceuticals. However, different from pharmaceuticals, personal care products are designed to be used directly on human body to change appearance, taste, and odor [60]. In recent years, a large number of personal care products and their transformed products have been found in wastewater treatment plants. Part of them would be converted into carbon dioxide and other harmless inorganic compounds. And part of them can be adsorbed by sedimentation sludge through surface chemical interaction. However, the others and their metabolized products escape the wastewater treatment plants and are more resistant to being degraded in the environment. The most possible emerging pollutants in personal care products include microplastics (e.g., polyvinyl chloride, polystyrene, polyethylene, and propylene) [61], fragrance pollutants (e.g., galaxolide, tonalide, iso-E-super, and exaltone) [62], insect repellents (e.g., icaridin, geraniol, thiabendazole, and diethyltoluamide) [63,64], and preservatives (e.g., methylparaben, propylparaben, rosmarinic acid, and carvacrol) [65]. For example, it was reported that the microplastics abundance in the river network of eastern China was up to 104.6 ± 5.6 particles/L in 2018–2019 [66]. Since microplastics are often used in a regular manner, trace amount of this pollutant may cause harm to humans, animals, and the environment.

### 2.3 Pesticides

Pesticides are reagents with specific physical and chemical properties, protecting farming from dangerous insects, weeds, and microorganisms. According to the target species, pesticides are conventionally classified into four classes: insecticides (e.g., aldicarb, malathion, avermectin, and rotenone) [67], herbicides (e.g., atrazine, clopyralid, alachlor, and oxyfluorfen) [68], fungicides (e.g., oxathiapiprolin, myclobutanil, chlorothalonil, and mandipropamid) [69], and bactericides (e.g., triadimefon, diclazone, methyltobuzin, and likrypton) [70]. Among them, insecticides and herbicides dominate 29.5% and 47.5% of total pesticide consumption, respectively. Pesticides have adverse impacts on short-term sickness to numerous cancers of humans, water quality, plants, and animals. However, it is very difficult to reduce their use, as the crops are vulnerable to pests and diseases, and their yields will decrease remarkably with the reduction of pesticides.

### 2.4 Perfluorinated compounds

Perfluorinated compounds are synthetic substances in which the hydrogen atoms in the alkyl molecular chain are saturated or substituted by fluorine atoms. Due to the high hydrophobic properties of fluorine atoms and high bonding energy of F–C bond, perfluorinated compounds exhibit superior stability. They have been widely used in consumer and industrial manufactures, such as pigments, paintings, and protective coatings [71]. However, perfluorinated compounds can persistently exist in environment and are hardly degraded. Nowadays, they are world-widely distributed and detected at concentrations from μg/L to ng/L [72]. According to the chemical structures, perfluorinated compounds can mainly be divided into perfluoroalkyl carboxylic acid (e.g., perfluorooctanoic acid, perfluorobutanoic acid, perfluorohexanoic acid, and perfluorononanoic acid), perfluoroalkyl sulfonic acid (e.g., perfluorooctanesulfonic acid, perfluorobutenesulfonate, perfluoropentanesulfonic acid, and perfluoro-nonanesulfonic acid), perfluorosulfonamides (e.g., perfluorooctane sulfonamide, perfluorobutyl sulfonamide, perfluoroethyl sulfonamide, and N-methyl perfluorobutane sulfonamide), and perfluorotelomer alcohol (e.g., 2-perfluorooctyl ethanol, 2-perfluorohexyl ethanol, 2-perfluorobutyl ethanol, and perfluoroalkyl ethanol). Among them, perfluorooctanoic acid and perfluorooctane sulfonate are the dominant perfluorinated compounds in terms of their concentrations and detection frequency [73]. Perfluorinated compounds can interfere with the endocrine system and alter the instinctive behavior of animals, posing a great threat to human beings [74]. Overall, pharmaceuticals, personal care products, and pesticides have been confirmed to affect humans and environment, yet they are still discharged into the immediate environment unregularly and carelessly. Therefore, it is stringent to remove these emerging contaminants for water body.

## 3 Carbon-based SACs

Developing scalable and controllable synthetic strategies for carbon-based SACs preparation is of significant importance to the industrialization of carbon-based SACs in persulfate-based AOPs. Due to its high surface energy, the isolated single metal atom always migrates and aggregates with each other, forming a nanoparticle or cluster [75]. Only when the interaction between a single metal atom and carbon carrier is strong enough to anchor each metal atom, carbon-based SACs can be obtained [76]. Therefore, the principles and rules for carbon-based SACs preparation lie in the construction of stable interaction between a single metal atom and carbon carrier. Recently, pyrolysis [77], defect/vacancy trapping [78], impregnation transfer [79], atomic layer deposition (ALD) [80], and other strategies [81] have been developed to prepare carbon-based SACs. The as-prepared catalysts have different chemical and electronic structures that can induce radical or nonradical pathway in persulfate-based AOPs. Characterizations of the structures of these carbon-based SACs can help us disclose the relationship between structures and catalytic pathways, the focus in this review, realizing the rational design of high-performance catalysts and ultimately industrial application.

### 3.1 Synthetic strategies of carbon-based SACs

#### 3.1.1 Pyrolysis

The pyrolysis strategy holds great promise in the mass-up production of highly active carbon-based SACs. Based on the chemical properties of carbon sources, this strategy can be classified into four categories: direct pyrolysis anchoring [77], pyrolysis of metal–organic frameworks (MOFs) [82], pyrolysis of nitrogen-rich organics [83], and pyrolysis of polymers [84]. Direct pyrolysis anchoring means direct thermal treatment to anchor surface bonded atomically dispersed metal atoms (e.g., Fe, Cu, Ni, and Co) on carbon carriers (e.g., graphene oxide, carbon nanotubes, and nitrogen-doped graphene). For example, Fei et al. dispersed transition metal atomically on graphene oxide through the coordination between transition metal atom and the surface functional group of graphene oxide [77]. Then, a series of atomic transition metals embedded in nitrogen-doped graphene with M−N_4_ moiety were prepared after thermal annealing [77]. Bi et al. reported the adsorption of Ni(II)-cyclam complex on nitrogen-doped graphene via π-π interaction and anchoring of Ni^2+^ after pyrolysis [85]. By using direct pyrolysis anchoring, atomically dispersed transition metals on nitrogen-doped carbon nanotubes with ultrahigh metal loading up to 20 wt% were also produced [86].

**Fig. 3:**
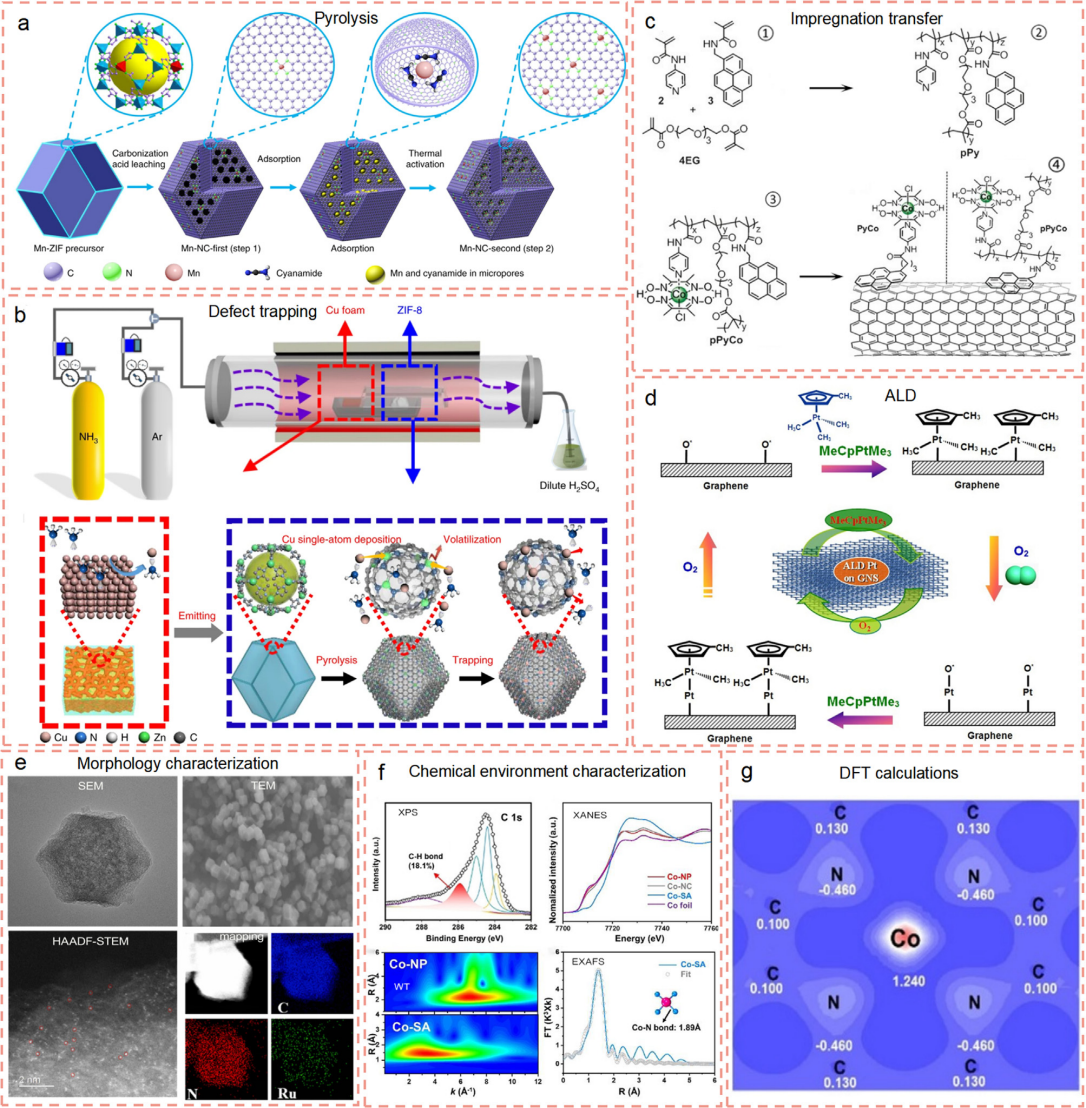
Construction and characterization of carbon-based SACs. (a) Schematic of atomically dispersed Mn–N_4_ site catalyst synthesis through pyrolysis (Reproduced with permission from ref. [87]. Copyright (2018) Springer Nature). (b) Schematic of preparation of Cu–SAs/N–C through defect trapping (Reproduced with permission from ref. [78]. Copyright (2018) Springer Nature). (c) Impregnation transfer of single atom Co in pyrenebearing cobaloxime copolymer to multiwall carbon nanotubes (Reproduced with permission from ref. [94]. Copyright (2018) Wiley-VCH). (d) Schematic illustrations of Pt atomic layer deposition (ALD) mechanism on graphene nanosheets (Reproduced with permission from ref. [80]. Copyright (2013) Springer Nature). (e) Scanning electron microscopy (SEM), transmission electron microscopy (TEM), aberration-corrected high-angle annular dark-field scanning transmission electron microscopy (HAADF-STEM), and mapping images of RuSA-N-C (Reproduced with permission from ref. [105]. Copyright (2023) Elsevier). (f) N 1s XPS, normalized Co K-edge X-ray absorption near edge structure (XANES), and the wavelet transform (WT) spectra of different samples, and corresponding the extended X-ray absorption fine structure (EXAFS) fitting curves of Co-SA in R space (inset is proposed Co-N_x_ site) (Reproduced with permission from ref. [106]. Copyright (2021) Wiley-VCH). (g) Valence electron density map of Co–N_2+2_ site in Co-SA (Reproduced with permission from ref. [106]. Copyright (2021) Wiley-VCH).

MOFs represent a new class of porous materials synthesized from the self-assemble of organic ligands and metal ions. As depicted in Fig. 3a, the copious organic ligands in MOFs can accommodate the single metal ions separately and themselves transform into nitrogen-doped graphene after pyrolysis. As a result, metal atoms dispersed nitrogen-doped graphene can be obtained by pyrolysis of MOFs. Meanwhile, carbon-based SACs produced by pyrolysis of MOFs have large surface areas and ordered pores, which are beneficial for the dispersion and stabilization of single metal atoms. Recently, different kinds of MOFs, such as ZIF-8, ZIF-67, and UIO-66, have been widely reported as precursors for the synthesis of carbon-based SACs. Among them, ZIFs are the most favorable bimetal MOFs for the synthesis of carbon-based SACs, in which the sacrifice metal zinc compound evaporates and the coordinated metal ions are atomically anchored. It was reported that Mn^2+^ [87] and Ni^2+^ [88] were crabbed by the organic ligands in ZIF-8, converting them into Mn and Ni SACs via pyrolysis, respectively.

Due to the coordination between nitrogen-containing functional groups and metal ions, nitrogen-rich organics have been selected as low-cost precursors for carbon-based SACs preparation. During pyrolysis, nitrogen-rich organics can themselves convert into carbon as the carrier and simultaneously the doped N can anchor metal atom stability. In 2021, Cai et al. first pyrolyzed copper disodium ethylene diamine tetraacetic acid precursor at low-temperature and synthesized a carbon-dots-based SACs with unique CuN_2_O_2_ sites [83]. Expect for N, introduction of boron, sulfur, phosphorus, and other heteroatoms into precursors can even regulate the coordination environment of SACs and ultimately govern the catalytic performances. Gu et al. introduced boric acid as a boron precursor into glucose and ammonia system, giving a boron and nitrogen codoped ultrathin porous carbon-support single-atom nickel catalyst with a special NiN_4_B_2_C_x_ active site [89]. This NiN_4_B_2_C_x_ active site exhibited higher current density and better selectivity in CO_2_ reduction reaction than the NiN_4_ active site, since the enhanced adsorption of CO_2_ and COOH intermediate, and decreased free energy barriers of CO_2_RR pathway.

Carbon-based SACs can also be prepared by one-step pyrolysis of polymers in which the C element can play a role of a carbon source, and N element can coordinate and stabilize metal atoms. Polymers, such as polyaniline, polydopamine, melamine–cyanurate polymer, and melamine–resorcinol–formaldehyde polymer [90], have been reported in the preparation of carbon-based SACs. Zheng and coworkers developed a family of single metal atoms (Fe, Co, Ni, and Cu) immobilized graphitized carbon materials by one-step *in-situ* pyrolysis of metalloporphyrin and melamine–cyanurate polymer [84]. Pan et al. constructed a Co–N_5_ site through coordination interaction between Co and N on melamine–resorcinol–formaldehyde polymer derived hollow N-doped porous carbon spheres [91]. Although the pyrolysis strategies have their own advantages, direct pyrolysis anchoring is facile, time-saving, and cost-effective without complex posttreatment, such as acid washing. Thus, it is recognized to be of high potential for facile and mass-up production of carbon-based SACs.

#### 3.1.2 Defect trapping

The prerequisite for carbon-based SACs preparation is stabilizing a single metal atom on a carbon carrier. Different from pyrolysis strategy, the defects trapping strategy relies on spatial confinement to grasp and isolate the metal atoms. It was demonstrated that the defect in ZIF-8 was capable of trapping Cu single atoms (Fig. 3b). Accompanying with meticulous control of Cu atom emitting, copper single atoms supported on nitrogen-doped carbon could be synthesized [78]. When the emission of Cu atom exceeded an upper limit, Cu nanoparticles were observed instead of single Cu atoms. Additionally, the property of defect could influence the formation of SACs. Cheng and coworkers selected defective graphene to deposit Pt [92]. However, only Pt clusters with an average size of approximately 0.80 nm could be obtained rather than Pt single atom.

#### 3.1.3 Impregnation transfer

The impregnation transfer is based on the covalent, π-π stacking, electrostatic, or coordination interactions between carbon materials and single-atom compounds [93]. Single atoms can be easily implanted on different carbon materials (activated carbon, graphene or carbon nanotubes) through impregnation into these materials. Sun et al. dropped HAuCl_4_ solution into aqua regia impregnated activated carbon and single-site Au/C catalysts were prepared after drying [79]. Through π-π stacking, the single atom Co in pyrenebearing cobaloxime copolymer was transformed into multiwall carbon nanotubes (Fig. 3c) [94]. This impregnation transfer strategy takes advantages of preserving the well-defined atomic structures of carbon carrier and regulating their electronic structures, while the metal loading amount is limited. The low metal amount causes a deficient of catalytic centers for high-performance catalysis.

#### 3.1.4 Atomic layer deposition

ALD is a thin-film growth technology in which the individual metal atom can deposit one-by-one under precise control. Under this circumstance, ALD merges as an effective method to prepare carbon-based SACs. Generally, ALD method involves the evaporation of metal source into reaction chamber and its deposition on substrate by an inert carrier gas. Fig. 3d illustrates that Sun and co-colleagues first employed ALD technique to fabricate single-atom Pt on graphene surfaces by precise control of the number of ALD cycles in 2013 [80]. In 2019, Yan et al. utilized ALD technology to fabricate single-atom Pt supported on the defective graphene [95]. Through adjusting the deposition cycles, the size of Pt composition can be tuned from the Pt single atom, Pt clusters to Pt nanoparticles. However, ALD technique is energy intensive and makes it difficult to prepare SACs with a high metal loading, which greatly hinders its practical application of carbon-based SACs in AOPs.

#### 3.1.5 Others

Ball milling and chemical vapor deposition (CVD) are emerging preparation strategies to fabricate carbon-based SACs. For high-energy ball milling, the bonds of chemicals can be easily broken and then reconstruct with each other to form a new compound [96]. A series of single-atom-anchored graphene nanosheets with M−N_4_ (M = Mn, Fe, Co, Ni, or Cu) active sites was first developed by Bao’s group, through ball milling the mixture of metal phthalocyanine and graphene nanosheets [81]. However, in this method, the morphologies and crystal facets of carbon-based SACs are uncontrollable. For CVD method, the gaseous organic molecules can interact with the metal (e.g., copper and nickel) film or foil substrate, growing carbon-based SACs [97]. Although CVD is capable of preparing carbon-based SACs with a large area, its low yield greatly limits the practical application.

Based on above discussion, it is found that carbon-based SACs can be prepared via various strategies. However, there are some hard issues to be conquered before their final practical application in persulfate-based AOPs [28]: (i) Cutting production costs and increasing the productivities; (ii) understanding the formation mechanism, regulating the structures of active sites (coordination number, anions and oxidation state) and increasing metal loading (without aggregation); (iii) improving the stability of structures and preventing metal leaching.

### 3.2 Characterization of carbon-based SACs

#### 3.2.1 Chemical structure carbon-based SACs

Similar to bulk or nanomaterials, scanning electron microscopy (SEM) and transmission electron microscopy (TEM) have been used to characterize the morphologies of carbon-based SACs [98,99]. From the SEM and TEM images in Fig. 3e, it is observed that single Ru atoms dispersed on N-doped carbon (RuSA-N-C) exhibit typical rhombic dodecahedron shape with uniform size distribution. Conventionally, TEM often has a higher resolution in imaging than SEM. However, it is still difficult to “visualize” the single atom, since its image contrast relies on the diffraction and phase effects. In contrast, the aberration-corrected high-angle annular dark-field scanning transmission electron microscopy (HAADF-STEM) is powerful to directly “visualize” the atomically dispersed active sites in various SACs [100]. The single Ru atoms in RuSA-N-C are directly visualized in the HAADF-STEM image shown in Fig. 3e. Moreover, HAADF-STEM can be used to characterize the coordination environment and atom distances of each other.

As a sensitive surface analysis technology, X-ray photoelectron spectroscopy (XPS) can also be used to identify the chemical environment of surface elements, as well as elemental compositions [101]. The C–H bond is detected in the C 1s spectrum implying the existence of Co–N_2+2_ configuration in carbon-based Co SACs (Fig. 3f). However, this evidence is not adequate to support the conclusion, as XPS only provides surface information of carbon-based SACs, rather than the inner or overall information of the materials [102]. On the other hand, the XPS signal of metal elements is usually very weak, as there is a very low loading amount in the carbon-based SACs. In comparison, X-ray absorption spectroscopy (XAS) is very sensitive to the measurement of local chemical environment of carbon-based SACs, which can complement the XPS results. In general, XAS comprises two categories: the extended X-ray absorption fine structure (EXAFS) and X-ray absorption near edge structure (XANES). The EXAFS evaluates the local bonding environments, including coordination number, interatomic distance, and chemical bonding in SACs [103]. For XANES, it provides the geometric and electronic structures of SACs [104]. Combining the EXAFS and XANES analyses, we can minimize uncertainties of human operations and improve the precision of results. Finally, we concluded the existence of a Co–N_2+2_ configuration with a Co–N bond of 1.89 Å in carbon-based Co SACs (Fig. 3f).

#### 3.2.2 Electronic structure of carbon-based SACs

The catalytic performances of carbon-based SACs in persulfate-based AOPs are also determined by the electronic structures of the atomically dispersed active sites. Density functional theory (DFT) calculations directly reflect the electronic features of carbon-based SACs and predict the interaction between single atom active site and reactant [107]. Mi and coworkers calculated the electron density of Co–N_2+2_ sites, pointing to the electron-deficient property of Co center (Fig. 3g) [108]. Further, they employed DFT calculations to reveal the ^1^O_2_ generation mechanism at Co–N_2+2_ sites during PMS activation. DFT calculations results indicated an energy barrier for the decomposition of PMS via an O–O or O–H bond cleavage to generate radicals. Instead, the electrons transfer from PMS to Co–N_2+2_ sites, resulting in the generation of ^1^O_2_. DFT calculations, are helpful to understand the mechanism in-depth and provide guidelines for the rational design of carbon-based SACs at the atomic and molecular scales.

## 4 Application of carbon-based SACs in persulfate-based AOPs

Recently, carbon-based SACs have been regarded as the next frontier in heterogeneous PDS/PMS activation for the degradation of organic pollutants [35,109]. By activation of PDS/PMS, different ROS can be produced, inducing radical pathways, nonradical pathways, and their combination in AOPs. The types of ROS mainly determine the catalytic performance and applicability of PDS/PMS activation [110]. Herein, we summarize the catalytic reactions in view of the types of their generated ROS and deliberately discuss the relationship between ROS generation and the single metal atom active sites (Table 1).

### 4.1 Radical pathway

#### 4.1.1 Radical pathway induced by carbon-based Fe-SACs

**Fig. 4:**
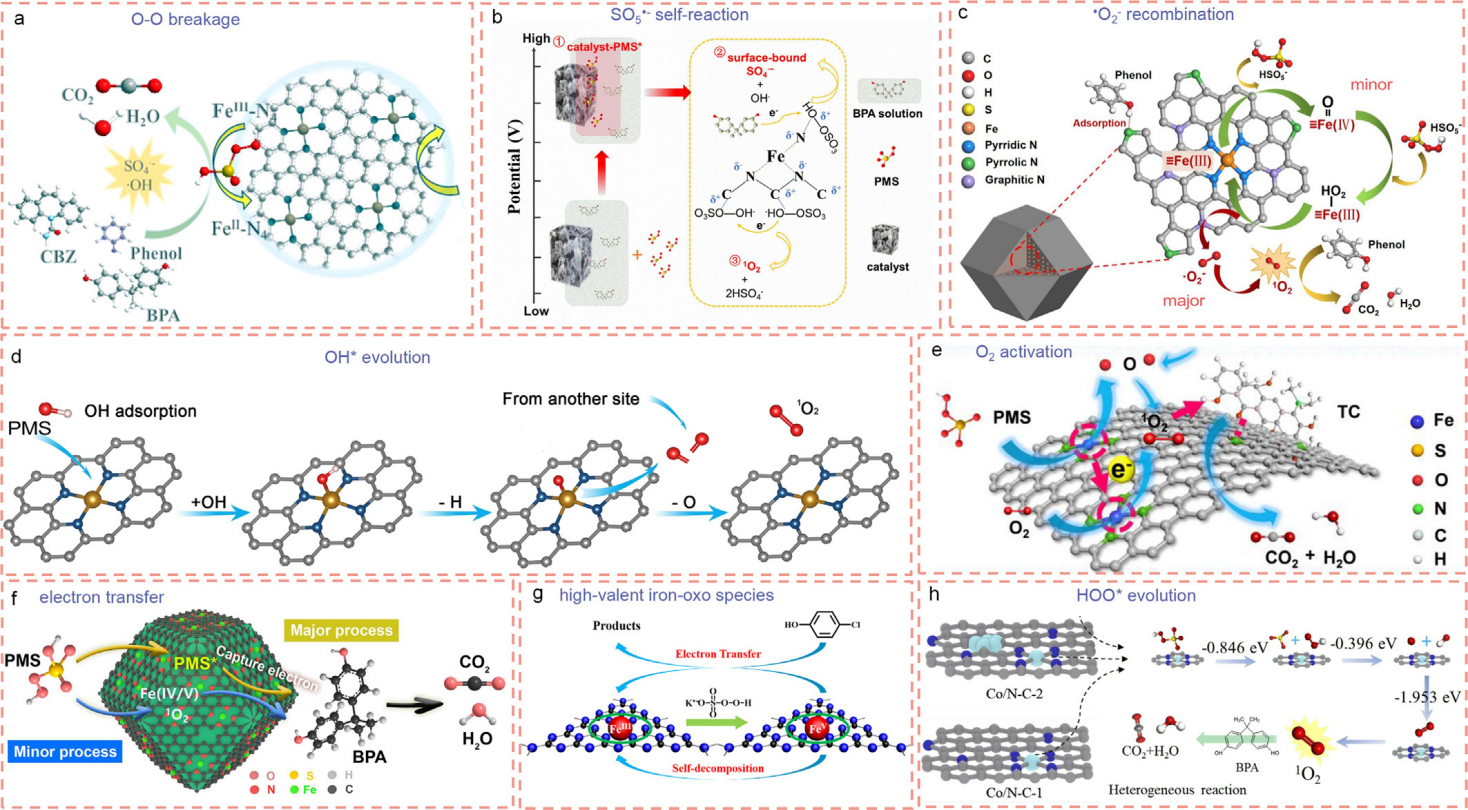
Generation mechanisms of different reactive oxidant species at single-atom active sites. (a) Schematic illustration of SO_4_^•-^ and ^•^OH radicals generation mechanism over Fe(II)–N_4_ active sites during PMS activation (Reproduced with permission from ref. [115]. Copyright (2022) American Chemical Society). Proposed (b) SO_5_^•-^ self-reaction mechanism (Reproduced with permission from ref. [135]. Copyright (2021) Elsevier), (c) ^•^O_2_^-^ recombination mechanism (Reproduced with permission from ref. [130]. Copyright (2020) Elsevier), (d) OH∗ evolution mechanism (Reproduced with permission from ref. [133]. Copyright (2021) Wiley-VCH), and (e) O_2_ activation mechanism (Reproduced with permission from ref. [146]. Copyright (2023) American Chemical Society) for ^1^O_2_ generation by activation of PMS via carbon-based Fe SACs. (f) Proposed electron transfer mechanism in the PMS + FeSA-N/C-20 system ( Reproduced with permission from ref. [141]. Copyright (2021) Elsevier). (g) Degradation Mechanism of 4-chlorocatechol by high-valent iron-oxo species (Reproduced with permission from ref. [144]. Copyright (2018) American Chemical Society). (h) Mechanism of PMS activation in Co/N–C/PMS systems (Reproduced with permission [157]. Copyright (2022) Elsevier).

Carbon-based Fe-SACs have been widely reported in the application of persulfate-based AOPs, due to high mineral reserves, low prices, and low toxicity of Fe than other transitional metals (Co, Ni, and Mn) [111,112]. Compared with the Fe nanoparticles anchored on the hierarchical porous biochar (nano-Fe/MC), its counterpart, carbon-based Fe-SACs, exhibited 33.2-fold enhancement in PMS activation to degrade phenol [113]. The extraordinary catalytic performance of carbon-based Fe-SACs is attributed to the more efficient radical generation than that induced by Fe nanoparticles. It was reported that Fe single atoms anchored nitrogen-doped graphene (Fe-SA-NG) with Fe–N_4_ active sites could activate PDS/PMS to generate SO_4_^•-^ and ^•^OH (Fig. 4a) [36,[114], [115], [116], [117]]. The enhanced radical generation mechanisms at Fe–N_4_ active site in Fe-SA-NG are summarized as follow: (i) The increased density of states around Fermi level of Fe single-atom active sites in Fe-SA-NG promote the electron transfer from Fe single atom site to PMS for SO_4_^•-^ and ^•^OH generation. (ii) Fe single-atom active sites are electron-rich as demonstrated experimentally and theoretically. They are more likely to donate electrons to PMS and promoted the dissociation of PMS via O–O bond cleavage to generate SO_4_^•-^ and ^•^OH [114]. (iii) XAS and ^57^Fe Mössbauer spectroscopy reveals that the 3d_z_^2^ orbital of high-spin Fe(II) (S = 2) is occupied by a single electron [115]. The generation of SO_4_^•-^ and ^•^OH radicals through a one-electron transfer process at Fe(II)-pyridinic N_4_ active sites is thermodynamically favorable [118].

The unsaturated Fe–N_4_ active sites possess fast reaction kinetics for SO_4_^•-^ and ^•^OH generation because of their strong coordination capability toward PMS [119]. However, the uncoordinated 3d electrons in Fe metal atoms are adverse to the desorption of the negative bisulfate group generated by PMS [120]. It requires high energy to ensure a high-speed radical generation cycle at Fe–N_4_ active sites, by competing for the adsorption of PMS with generated negative bisulfate intermediates. To overcome this problem, Liu’s group developed a carbon nitride nanosheet-supported single-atom Fe catalyst with unique single-atom FeN_5_ active sites [121]. The FeN_5_ active sites can balance PMS adsorption and bisulfate intermediates desorption, regenerating the active sites for a more efficient radical generation. Meanwhile, the as-synthesized catalyst possesses a high Fe loading amount, up to 16.64 wt%. This improves the density of active sites for more SO_4_^•-^ and ^•^OH generation to degrade organics.

The intrinsic properties of Fe single atoms active sites are determinal factors toward SO_4_^•-^ and ^•^OH generation, while the carbon carrier could also influence the reaction processes. Gao et al. pointed out that the pyridinic N in Fe–N_4_ active sites could induce positive charge distribution of neighboring C atoms, enhancing the adsorption of PMS to dissociate into radical species [114]. In contrast, Xiong et al. reported that the graphitic N rather than pyridinic N in carbon-based Fe-SACs is an adsorptive site for PMS, promoting the radical processes [36]. Further research is expected to identify the roles of carbon carriers in radical generation in persulfate-based AOPs.

#### 4.1.2 Radical pathway induced by carbon-based Co-SACs

Co^2+^ is the most effective metal ion for PDS/PMS activation to degrade organics [122]. Therefore, studies about carbon-based Co-SACs preparation and application have naturally been carried out [[123], [124], [125], [126], [127], [128]]. In persulfate-based AOPs, the reported coordination structures in carbon-based Co-SACs that induce radical generation mainly include Co–O_2_ [127], Co–N_4_ [124], and Co–N_3_ [126]. For Co–O_2_ active site, the Co single atom is incorporated into two-dimensional graphene oxide flakes [127]. The positively charged Co single atom facilitates the adsorption of negatively charged PMS molecule and transportation of π electrons of graphene oxide flakes to adsorbed PMS, resulting in the cleavage of O–O bond in PMS to produce SO_4_^•-^ and ^•^OH. For Co–N_4_ active site, its reaction mechanism is similar to that in Co–O_2_ active site. The Co single atom in Co–N_4_ active site exhibits positive charge [124], enhancing the adsorption of PMS on the Co single atom to decompose via a radical pathway. For Co–N_3_ active site, a similar reaction mechanism was also proposed [125,126].

Liang et al. reported that the low coordinated Co–N_3_ active sites exhibited 1.31 times higher activity than the Co–N_4_ active sites for bisphenol A degradation via PDS activation [126]. PDS is more favorable to dissociate into SO_4_^•-^ and ^•^OH at Co–N_3_ active sites than at Co–N_4_ active sites. The adsorption energy of PDS on Co–N_3_ active site is much higher than that on Co–N_4_ active site. Further, the Bader charge analysis pointed out that much more electron transport from the single-atomic Co site in Co–N_3_ active sites (0.102 e) toward the PDS molecule than that in Co–N_4_ active site (0.042 e), which is more conducive to accepting electron for PDS over Co–N_3_ active site. However, Zhu et al. reported that the Co–N_3_ active sites were less effective in PMS activation to give radicals than the CoN_4_ active sites [125]. A higher energy barrier should be overcome by Co–N_3_ + PMS complex before receiving electrons than Co–N_4_ + PMS complex. The difference might be due to the structure different between PDS and PMS, which might be able to affect the electronic structure of single atom active sites after adsorption.

#### 4.1.3 Radical pathway induced by carbon-based other metal SACs

Expect for Fe and Co, the other single metal atom active site that can activate PDS/PMS to totally generate SO_4_^•-^ and ^•^OH is rare [129]. By pyrolysis of Mn-doped MOFs, isolated single Mn atom on N-doped porous carbon matrix (Mn–ISAs@CN) was synthesized and first reported to generate radicals via activation of PMS to degrade various emerging contaminants [129]. The as-synthesized Mn–ISAs@CN catalyst endows Mn–N_4_ active sites, which have a moderate adsorption energy for PMS and a desorption energy for the intermediates. This enables the effective dissociation of PMS and generation of SO_4_^•-^ and ^•^OH after one-electron transfer from MnN_4_ active sites to PMS. Overall, in PDS/PMS activation, the electron transfer direction determines the dissociation pathways of PDS/PMS, namely radical or nonradical. For radical pathway, PDS/PMS should accept electron from carbon-based metal-SACs, decomposing via breakage of O–O to generate SO_4_^•-^ and ^•^OH. The adsorption of PDS/PMS on a single metal atom active site limits the speed for a radical generation.

### 4.2 Nonradical pathway

#### 4.2.1 Nonradical pathway induced by carbon-based Fe-SACs

Different from the radical pathway in which SO_4_^•-^ and ^•^OH are responsible for organics degradation, the nonradical pathway often includes ^1^O_2_ generation [[130], [131], [132], [133], [134], [135], [136], [137], [138], [139]], electron transfer [140,141], and high-valent iron-oxo species [[142], [143], [144], [145]]. As an endothermic reaction, the free energy of ^1^O_2_ generation via PDS/PMS activation is calculated to reduce remarkably to 0.28 eV at Fe–N_4_ active sites than that at nitrogen-doped graphene (0.97 eV) [135]. This indicates that ^1^O_2_ generation is easier to generate at Fe–N_4_ active sites. Carbon-based Fe-SACs with Fe–N_4_ active sites are reported to produce ^1^O_2_ by activation of PDS/PMS through three different mechanisms including SO_5_^•-^ self-reaction [134,135,137], ^•^O_2_^-^ recombination [130,136], OH^∗^ evolution [133], and O_2_ activation [146]. For the SO_5_^•-^ self-reaction mechanism shown in Fig. 4b, the terminal O of PMS is adsorbed on Fe–N_4_ active sites, promoting the oxidization of PMS to SO_5_^•-^ through the loss of the H atom [134]. Then, SO_5_^•-^ reacts with each other to produce ^1^O_2_, due to the low activation energy and high reaction rate (Eqs. 1–3).

(1) HSO_5_^-^ → SO_5_^•-^ + H^+^ + e^-^

(2) SO_5_^•-^ + SO_5_^•-^ → S_2_O_8_^2^^-^ + ^1^O_2_

(3) SO_5_^•-^ + SO_5_^•-^ → 2SO_4_^2^^-^ + ^1^O_2_

For the ^•^O_2_^-^ recombination mechanism, it was proposed that the generation of ^1^O_2_ was related to the ^•^O_2_^-^ intermediate [130,136]. As illustrated in Fig. 4c, the Fe–N_4_ active sites are electron deficient and accept electrons from PMS, producing Fe (Ⅲ)-O-O-SO_3_ complex (Eq. 4) [130]. The Fe (Ⅲ)-O-O-SO_3_ complex experiences heterolytic cleavage of the O–O bond, reaction with another PMS molecule, and fast deprotonation process to give ^•^O_2_^-^ (Eqs. 5–7). Finally, ^•^O_2_^-^ radicals recombine to generate ^1^O_2_ for organics degradation (Eq. 8). Due to the fact that the overall process is thermodynamically favorable and has a total exothermic energy of 6.02 eV, this mechanism is believed to be reasonable.

(4) Fe(Ⅲ) + HSO_5_^-^ + H_2_O → [Fe(Ⅲ)-O-O-SO_3_]^+^ + H^+^

(5) [Fe(Ⅲ)-O-O-SO_3_]^+^ → Fe(Ⅳ) = O + SO_4_^•-^

(6) Fe(Ⅳ) = O + HSO_5_^-^ → Fe(Ⅲ)-HO_2_ + SO_4_^2^^-^

(7) Fe(Ⅲ)-HO_2_ → Fe(Ⅲ) + ^•^O_2_^-^ + H^+^

(8) 2^•^O_2_^-^ + 2H_2_O → H_2_O_2_ + 2OH^-^ + 2^1^O_2_

For the OH∗ evolution mechanism, Gao et al. showed that PMS was adsorbed on the Fe–N_4_ active sites by a single O atom of the SO_4_^-^ side and subsequently divided into OH^∗^ and SO_4_^∗^ moieties [133]. The formed OH^∗^ moiety transformed and absorbed on the Fe–N_4_ active sites to produce ^1^O_2_ by thermodynamically favorable desorption of O. The ^1^O_2_ generation pathway was concluded to be: PMS→OH^∗^→O^∗^→^1^O_2_ (Fig. 4d). For the O_2_ activation mechanism, Yang et al. demonstrated that the curved Fe–N_4_ active sites could directly transfer electron to O_2_ molecule and generate the ^1^O_2_ (Fig. 4e), due to the compressive strain of the curved Fe–N_4_ active sites with a higher energy level of Fe 3d_z_^2^ orbital [146].

Except for ^1^O_2_, emerging contaminants can also be degraded by electron transfer, another nonradical pathway, during PDS/PMS activation. Xu’s group [140] and Yin’s group [147] reported the electron transfer mechanism for the degradation of o-phenylphenol and bisphenol A in PMS activation, respectively. It was elucidated that the introduction of Fe single atom into a carbon carrier can promote its electron-donating ability. When the O–O bond in PMS is adsorbed on Fe–N_4_/FeN_4_O_1_ active sites, the electron is accumulated around Fe–O_2_ bond and then ulteriorly triggers the electron shuttling in organics degradation (Fig. 4f) [141].

The high-valent iron-oxo species are special “oxidants” that are produced from the activation of PDS/PMS at carbon-based Fe-SACs. Fe(IV)=O [142,143,148] and Fe(V)=O [144,145] are the most commonly reported high-valent iron-oxo species generated via PMS activation by carbon-based Fe-SACs. The valence states of original Fe centers in the carbon-based Fe-SACs greatly affect the types of high-valent iron-oxo species [149]. Usually, high-spin Fe(III)–N_4_ active sites can easily overcome the energy barrier for Fe(V)=O complex formation than Fe(II)–N_4_ active sites. Therefore, Fe(III) active sites tend to transform into Fe(V)=O via Eq. 9 during reaction (Fig. 4g) [144,145,150]. Sometimes, Fe(IV)=O can also be produced via Eqs. (4), (5) [143]. While for Fe(II)–N_4_ active sites, it first forms a surface complex of Fe(II)OOSO_3_ via coordination with PMS (Eq. 10) and then the Fe(II)OOSO_3_ heterolyzes at the O–O bond to generate reactive Fe(IV)=O (Eq. 11) [142].

(9) Fe(III) + S_2_O_8_^2^^-^ → Fe(V) + 2SO_4_^2^^-^

(10) Fe(III) + HSO_5_^-^ → Fe(II)–O–O–SO_3_ + H^+^

(11) Fe(II)–O–O–SO_3_ → Fe(Ⅳ) = O + SO_4_^2^^-^

#### 4.2.2 Nonradical pathway induced by carbon-based Co-SACs

Generally, carbon-based Co-SACs can degrade organic pollutants via a nonradical pathway including ^1^O_2_ generation, electron transfer, and high-valent Co species, during the activation of PDS/PMS [131]. The ^1^O_2_ generation mechanisms, to some extent, are similar to that induced by carbon-based Fe-SACs. They consist of SO_5_^•-^ self-reaction [35,[151], [152], [153], [154], [155]], OH^∗^ evolution [156], and HOO^∗^ evolution [157]. Similar to the generation of ^1^O_2_ at Fe–N_4_ active sites, the terminal O of PMS is first adsorbed on Co–N_4_ active sites and decomposes to SO_5_^•-^ intermediate and then followed by SO_5_^•-^ rapidly self-reacts to ^1^O_2_ [154]. Meanwhile, Li and colleagues found that the adsorption of organic molecules by a pyrrolic N site adjacent to Co single atom can shorten the migration distance between radical and substrates, leading to a higher catalytic performance, even much higher than the benchmark homogeneous (Co^2+^) and nanoparticulate (Co_3_O_4_) catalysts [35]. Compared with Co–N_4_ active sites, Co–N_2+2_ active sites can save 0.87 eV for PMS adsorption [106], which is more energy favorable to produce SO_5_^•-^ intermediate for ^1^O_2_ generation [106,158]. In addition, doping of P [153] or Si [159] atoms into Co–N_4_ active sites can concentrate the electron density and electron delocalization around the Co single atom centers, accelerating electron transfer from Co to PMS molecules for higher efficient ^1^O_2_ generation. The resultant zeolitic imidazolate framework-based catalyst (Co–N_3_P) exhibited a 2.5 times higher degradation rate than the undoped catalyst (Co–N_4_).

However, doping of O atom results in a different mechanism. Wang and coworkers found that O doping promoted PMS activation and reduced the energy barrier for O^∗^ generation, thus enhancing ^1^O_2_ generation at Co–N_3_O_1_ active sites via the optimized potential free energy diagrams: PMS→OH^∗^→O^∗^→^1^O_2_ [156]. On the other hand, Dai et al. pointed out that the cleavage of O–H bond in PMS to produce H^∗^ and SO_5_^∗^ needed 1.020 eV endothermic reaction energy, which was thermodynamically unfavorable [157]. After investigating the dynamic process of ^1^O_2_ generation, they believed that the generation of ^1^O_2_ was likely to experience the following steps: PMS→HOO^∗^→O^∗^→^1^O_2_ (Fig. 4h), due to the low-energy barrier of each step.

On the other hand, adsorption of PMS on Co single-atom active sites can form a “donor–acceptor complex,” which transfers an electron to organic pollutants for their degradation [132,160]. Compared with Fe, Mn, or Ni, Co possesses a high spin state that favors the overlap of its d orbitals with the oxygen-functional group of PMS and promotes spin-oriented electron transfer via Co–N_4_ active sites to degrade organic pollutants [132]. Additionally, it was reported that the unsaturated Co sites in Co–N_3_ exhibited a higher spin state and could enhance electron-donating ability, driving the valence transformation from Co(II) to Co(IV) [161]. The high-valent Co(IV) possesses a high oxidation ability to degrade organic pollutants.

#### 4.2.3 Nonradical pathway induced by carbon-based other metal-SACs

There are few reports regarding carbon-based Mn, Cu, or Ru-SACs for nonradical PDS/PMS activation [105,109,119,162]. Carbon-based Mn, Cu, or Ru-SACs can activate PDS/PMS to produce ^1^O_2_ over SO_5_^•-^ self-reaction mechanism. It was reported that Mn–N_4_ [109], Ru–N_4_ [105], and Cu–N_4_ [162] active sites favored the adsorption of PMS and donated an electron to PMS, giving SO_5_^•-^ intermediates for ^1^O_2_ generation. Interestingly, for the N-doped graphene-supported single copper atom catalyst (Cu_1_/NG), the distance between two Cu single atoms matched well with the molecular size of 10.13039/100011335PDS [119]. Due to the site distance effect, the catalytic performance of Cu_1_/NG showed almost two times higher turnover frequency than that of other Cu SACs without suitable Cu–Cu distance.

### 4.3 Radical and nonradical pathways

From the above discussion, we observe the radical or nonradical pathway during PDS/PMS activation initiated by carbon-based metal-SACs. However, due to the existence of multiactive sites or nonselective single metal-active sites, the co-occurrence of radical and nonradical pathways is also commonly reported during PDS/PMS activation by carbon-based metal-SACs [113,114,[163], [164], [165]]. For example, the Fe SACs deposited on *Myriophyllum aquaticum*-based biochar (ISA-Fe/MC) have dual active sites [113]. The single-atom Fe donates electron to PMS for the generation of SO_4_^•-^ and ^•^OH radicals, while the carbonyl group in ISA-Fe/MC is responsible for ^1^O_2_ production. Gao and coworkers demonstrated the similar phenomenon that the Fe-pyridinic N_4_ moiety contributes to the radical generation, and the C atoms adjacent to pyridinic N role as the active sites for ^1^O_2_ generation [114]. On the other hand, the Co-N_x_ [166] and Cu–N_4_ [164,165] active sites can simultaneously induce radical and nonradical pathways via PDS/PMS activation, respectively, without selective pathways. Overall, when an electron is transferred from a single metal atom active site to PDS/PMS, the radical pathway occurs. On the contrary, the nonradical pathway merges. However, the specific steps for radical or nonradical pathways are intricate and complex and are far from being elucidated. To address the problem, construction of carbon-based SACs with identical and clear structures is of high importance, which could help to reveal structure–performance relationship [167,168]. The essential understanding of the reaction mechanism is of certainty the landmark for a wide range application of carbon-base SACs in AOPs in the future [169,170].

## 5 Conclusion and perspectives

**Fig. 5:**
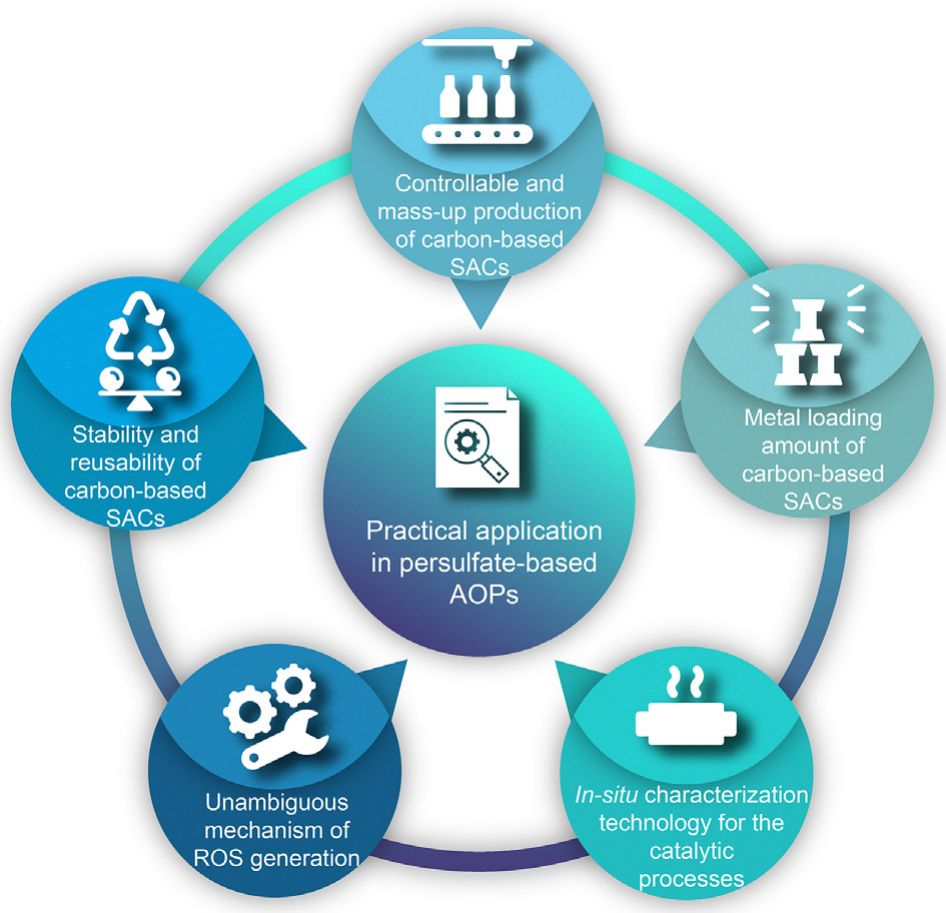
Recommended protocols for the application of carbon-based SACs in persulfate-based AOPs in future research.

In conclusion, we summarize recent investigations on emerging contaminants type, carbon-based SACs synthesis and characterization, and their environmental application. Particularly, we correlate the catalytic mechanism in persulfate-based AOPs with the structure of carbon-based SACs in detail from the aspect of ROS generation. Previously, tremendous carbon-based SACs have been well prepared and characterized, exhibiting superior catalytic performance to their nanoparticle/cluster counterparts for emerging contaminants degradation. The excellent catalytic performance benefit from the special geometric and electronic structure of single-atom active sites in carbon-based SACs. The single-atom active site can affect the adsorption of persulfate, regulating the electron transfer between active site and persulfate, and determining the cleavage of persulfate to generate different ROS. The difference in ROS ultimately affects the reaction pathway and catalytic performance. The radicals (^•^OH and SO_4_^•-^) exhibit higher oxidation ability to degrade emerging contaminants than the nonradical (^1^O_2_, e^-^, Fe(IV)=O and Fe(V)=O). On the contrary, the nonradical has a higher tolerance to the environment than the radical. Therefore, the simultaneous occurrence of radical and nonradical pathways induced by carbon-based SACs will guarantee highly efficient and versatile persulfate-based AOPs. In addition, carbon-based SACs reduce metal amount and anchor metal atoms firmly, which limits metal leaching and protects the environment, reverses the energy and maintain ecological balance. Although carbon-based SACs are believed to be of great potential in the application of persulfate-based AOPs, there are still some aspects that require more attention in future research (Fig. 5).

### 5.1 Controllable and mass-up production of carbon-based SACs

Although there are many synthetic methods, they still lack universality in the target construction of single-atom active sites. The principles and rules of material design and fabrication are still not universal, which makes the controllable synthesis of carbon-based SACs a great challenge. Meanwhile, the reported preparation strategies of carbon-based SACs, such as pyrolysis, defect/vacancy trapping, and other strategies, often require expensive equipment or high energy input. Thus, the mass-up production of carbon-based SACs is not economical and energy-saving. As the coordination structures greatly affect their catalytic activity, and the cost of materials preparation influences their budget, developing a general, inexpensive and mass-up methodology to synthesis carbon-based SACs needs further exploration before its industrialization application in persulfate-based AOPs.

### 5.2 Metal loading amount of carbon-based SACs

The loading amount of metal atoms is a pivotal parameter reflecting the number of active sites in carbon-based SACs and thus in determination of the catalytic performance in persulfate-based AOPs. In previous reports, the metal loadings of carbon-based SACs are usually less than 4 wt%. This greatly hinders the improvements of the catalytic performances and practical application. Increasing the metal precursor addition can result in the aggregation of metal atoms, giving nanoclusters or even nanoparticles. In order to increase the metal loading amount of carbon-based SACs, the following strategies might be effective: (i) Increase the number of defects or unsaturated coordination centers. For example, improve the N-doping level, providing more defect N sites to coordinate more metal atoms; (ii) Strengthen the intensity of coordination environment. For example, introduce other heteroatoms (expect for N) to create more firm interaction between metal atoms and carbon carriers; (iii) Form numerous small cavities to stabilize single metal atoms. For example, utilize more cavities in MOF to anchor more metal atoms. At last, it should be pointed out that different metal atoms have different interaction strength and loading dynamics with carbon carriers, which might result in great difference in their upper limited loading amount.

### 5.3 Stability and reusability of carbon-based SACs

The stability and reusability of the catalyst are important parameters to evaluate its industrial application. During the catalytic reactions, some of the degradation intermediates might be adsorbed on the surface of catalyst, resulting in the “poisoning” of catalyst. Meanwhile, the proportion of different N configurations in carbon-based SACs often changes remarkably after reaction. The graphitic N species are vulnerable to ROS and easily convert to pyrrolic N species after ROS attack. The above two phenomena greatly decrease the catalytic performance of carbon-based SACs in persulfate-based AOPs. Although the adsorbed intermediates and N configurations can be regenerated via pyrolysis, this will significantly increase the cost of catalytic reactions, hindering its industrial application. To solve these problems, the construction of a new single-atom active site with high tolerance to intermediates and ROS might be of potential. On the other hand, PMS presents a relatively strong acidity in the reaction solution, which is able to cause the leaching of metal atoms in catalysts. The metal leaching may both cause catalytic performance decrement and environmental pollution. Strengthening the coordination between the metal atoms and the carbon carriers is expected to effectively solve the metal leaching.

### 5.4 *In-situ* characterization technology for the catalytic processes

*In-situ* characterization technology can powerfully monitor the variation of generated ROS, structure change of catalyst, and interaction between catalyst and environment, probing the dynamic processes of the catalytic reaction. This characterization technology is beneficial for establishing the links between material structure and catalytic property. However, it has rarely been applied in the characterization of persulfate-based AOPs. The difficulty is ascribed to the very rare powerful ex-situ characterization methods for carbon-based SACs, not to mention the *in-situ* characterization technology. Although *in-situ* characterization technology, such as *in-situ* differential electrochemical mass spectrometer (*in-situ* DEMS), *in-situ* electron paramagnetic resonance (*in-situ* EPR), *in-situ* scanning electrochemical microscopy (*in-situ* DEMS), *in-situ* Raman spectroscopy, and *in-situ* XAS, have been widely applied in the study of mechanism of different catalytic reactions, most of them cannot directly reflect the interaction between catalysts and organic pollutants at atomic level. Only *in-situ* XAS can directly characterize the changes of atomically dispersed active sites. However, it was too expensive and rare to be obtained. Therefore, develop a facile and universal *in-situ* characterization technology is essential to the discovery of the catalytic processes. Given the high sensitivity, facile operation, and nondestructiveness, the spectroscopic technology is looking forward to being applied in the *in-situ* characterization of catalytic processes, establishing the relationship between different optical signals and coordination structures.

### 5.5 Unambiguous mechanism of ROS generation

In persulfate-based AOPs, the ambiguous ROS generation mechanism can help design and synthesis carbon-based SACs with target active sites, taking the advantage of radical and nonradical processes to meet the requirement of industrial application. Until now, it has not been clarified due to the limitation of synthesis and characterization of carbon-based SACs, and the lack of *in-situ* characterization technology for the catalytic processes. Although various synthetic strategies are reported, few of them can tune the interaction between the metal center and carrier. Thus, the effect of carriers on the chemical and electronic structures of carbon-based SACs cannot be systematically studied, making the mechanism of ROS generation difficult to be disclosed. On the other hand, the coordination structures of carbon-based SACs can be characterized by XAS, while the electronic structures are usually revealed by theoretical calculations instead of experiments. The potential discrepancies between theoretical and experimental results weaken the reliability of proposed ROS generation mechanism. In addition, lack of *in-situ* characterization technology also hinders the disclosure of reaction mechanism. More evidences are needed to demonstrate the mechanism, which is helpful to the industrial application of carbon-based SACs in the future.

## Declaration of competing interests

The authors declare no conflicts of interest.

## List of abbreviations

SACs: single-atom catalysts
AOP: advanced oxidation processes
ROS: reactive oxidant species
^•^OH: hydroxyl radical
SO_4_^•^^-^: sulfate radical
^•^O_2_^-^: superoxide radical
^1^O_2_: singlet oxygen
PDS: peroxydisulfate
PMS: peroxymonosulfate
CBZ: carbamazepine
BPA: bisphenol A
ZIF: zeolitic imidazolate framework
DEET: diethyltoluamide
PFOA: perfluorooctanoic acid
PFBA: perfluorobutanoic acid
PFHxA: perfluorohexanoic acid
PFNA: perfluorononanoic acid
PFOS: perfluorooctanesulfonic acid
PFBS: perfluorobutenesulfonate
PFPeS: perfluoropentanesulfonic acid
PFDS: perfluorononanesulfonic acid
PFOSA: perfluorooctane sulfonamide
PBSA: perfluorobutyl sulfonamide
PESA: perfluoroethyl sulfonamide
N-MeFBSA: N-methyl perfluorobutane sulfonamide
8:2 FTOH: 2-perfluorooctyl ethanol
6:2 FTOH: 2-perfluorohexyl ethanol
4:2 FTOH: 2-perfluorobutyl ethanol
FSO: perfluoroalkyl ethanol
ALD: atomic layer deposition
MOFs: metal–organic frameworks
EDTA: ethylene diamine tetraacetic acid
CVD: chemical vapor deposition
SEM: scanning electron microscopy
TEM: transmission electron microscopy
HAADF-STEM: high-angle annular dark-field scanning transmission electron microscopy
XPS: X-ray photoelectron spectroscopy
XAS: X-ray absorption spectroscopy
EXAFS: X-ray absorption fine structure
XANES: X-ray absorption near edge structure
WT: wavelet transform
DFT: density functional theory
Cu_1_/NG: N-doped graphene-supported single copper atom catalyst
ISA-Fe/MC: Fe single-atom catalysts deposited on *Myriophyllum aquaticum*-based biochar
